# Identification and Analysis of Differential miRNAs in PK-15 Cells after Foot-and-Mouth Disease Virus Infection

**DOI:** 10.1371/journal.pone.0090865

**Published:** 2014-03-27

**Authors:** Ke-Shan Zhang, Yong-Jie Liu, Han-Jin Kong, Wei-Wei Cheng, You-Jun Shang, Hong Tian, Hai-Xue Zheng, Jian-Hong Guo, Xian-Tao Liu

**Affiliations:** State Key Laboratory of Veterinary Etiological Biology, National Foot and Mouth Disease Reference Laboratory, Lanzhou Veterinary Research Institute, Chinese Academy of Agricultural Sciences, Lanzhou, China; George Mason University, United States of America

## Abstract

The alterations of MicroRNAs(miRNAs) in host cell after foot-and-mouth disease virus (FMDV) infection is still obscure. To increase our understanding of the pathogenesis of FMDV at the post-transcriptional regulation level, Solexa high-throu MicroRNAs (miRNAs) play an important role both in the post-transcriptional regulation of gene expression and host-virus interactions. Despite investigations of miRNA expression ghput sequencing and bioinformatic tools were used to identify differentially expressed miRNAs and analyze their functions during FMDV infection of PK-15cells. Results indicated that 9,165,674 and 9,230,378 clean reads were obtained, with 172 known and 72 novel miRNAs differently expressed in infected and uninfected groups respectively. Some of differently expressed miRNAs were validated using stem-loop real-time quantitative RT-PCR. The GO annotation and KEGG pathway analysis for target genes revealed that differently expressed miRNAs were involved in immune response and cell death pathways.

## Introduction

MicroRNAs (miRNAs) are small 21–24 nucleotide (nt) non-coding RNAs that play an important role in the post-transcriptional regulation of gene expression [1], [2]. Mature miRNAs are not directly produced by gene transcription, they are processed from long primary miRNA (pri-miRNA) precursors. Now they are known to play a key role in biological activities at the molecular level [3]–[5]. Research indicates that miRNAs not only bind to sites in the coding region and 5′UTR to regulate genes but may also combine with the 3′UTR of target mRNAs involved in development, fat metabolism, apoptosis, virus defense, and cell proliferation [6], [7]. Recent studies mainly focused investigating miRNA function in human disease and primarily relate to the use of miRNAs as disease biomarkers to monitor drug efficacy. In veterinary science, the stability of miRNAs along with their ability to regulate gene expression is beneficial in developing breeding programs for disease resistance in livestock [8].

MiRNAs offer an immense opportunity not only to develop novel biomarkers and therapeutics but also to understand the intricacies of host-pathogen interaction, and gain possible insights into viral oncogenesis, latency and tropism [9]. Researches showed that miRNAs play a critical role in host-virus interaction [10], [11] Indeed, host miRNAs have a substantial effect on viral evolution and potentially regulate the tissue tropism of viruses in vivo [10]. Viruses encode their own miRNAs in the miRNA-induced gene-silencing pathway [4]. For example, baculovirus-encoded miRNA (bmnpv-miR-1) suppresses its host miRNA biogenesis by regulating the exportin-5 cofactor Ran [12]. Enterovirus-induced miR-141 contributes to shutoff host protein translation by targeting the translation initiation factor eIF4E [13]. The host miRNA miR-122 is an indispensable factor in supporting hepatitis C virus (HCV) replication [14]. MiRNA-141 represses HBV replication indirectly by targeting PPARA. Human miRNA hsa-miR-296-5p suppresses *Enterovirus* 71 replication by targeting the viral genome [15]. Additionally, miR-125b and miR-223 directly target human immunodeficiency virus type 1 (HIV-1) mRNA, and miR-198 modulates HIV-1 replication indirectly by repressing the expression of ccnt1 gene [16], [17].

In recent years, the emergence of Solexa and 454 high-throughput sequencing technologies have enabled the direct sequencing of miRNAs to be a highly sensitive and and efficient method to study miRNAs in different species [18]–[21], although only about 250 pig miRNAs have been identified. Parameswaran reported virus-derived miRNAs profile in cells infection with dengue virus, vesicular stomatitis virus, polio virus, hepatitis C virus and West Nile virus [22]. Deep sequencing revealed HIV-encoded miRNAs modulate cellular and viral gene expression [23]. Using the technique, other researchers found that pseudorabies virus (PRV) encoded miRNAs that play significant role in virus-cell interaction. These miRNAs are mainly involved in complex cellular pathways including cell death, immune system, and metabolism [24]. Following sequencing, northern blot hybridization and highly sensitive stem-loop RT-PCR are generally used to identify mature miRNAs and confirm changes in expression levels of miRNA based on known unique sequences [25].

Foot-and-mouth disease (FMD) is a highly infectious disease of cloven-hoofed domestic and wild animals [26]. Seven serotypes (A, O, C, Asia 1, and South African Territories 1, 2, and 3) have been identified, and multiple subtypes occur within each serotype [26]–[28]. The etiologic agent, FMD virus (FMDV) belongs to *Aphthovirus genus* of the *Picornaviridae* family. FMDV consists of a single-stranded, plus-sense RNA genome of approximate 8,500 bases surrounded by an icosahedral capsid composed of 60 copies each of four structural proteins VP1-4 (termed 1D, 1B, 1C, and 1A). However, the mechanism of FMDV infects host cells in post-transcriptional regulation is unclear.

The focus of this study is differential expressed miRNAs between FMDV-infected and uninfected PK-15 cell groups. PK-15 cell line was purchased from Center for Type Culture Collection(CTCC, Wuhan, China). To gain further insights into the potential role of miRNAs during FMDV infection of host cells, small RNA from PK-15 cell libraries of individual FMDV infection and uininfected groups were constructed. Solexa highthroughput sequencing technique and bioinformatics for sequencing were integrated and data processing to:

1. obtain the miRNA expression profiles between the two groups of cells;
2. identify the novel and differentially expressed miRNAs;
3. define the regulatory network between miRNAs and mRNA; and
4. determine the regulatory mechanisms of miRNAs involved in the FMDV infection.

The results updated the pig miRNA database to better understand the molecular mechanism of FMDV infection. Our findings layed a new foundation for identifying the biomarkers associated with the FMDV infection.

## Results

### Small RNA Library Construction and Solexa Sequencing

**Figure 1:**
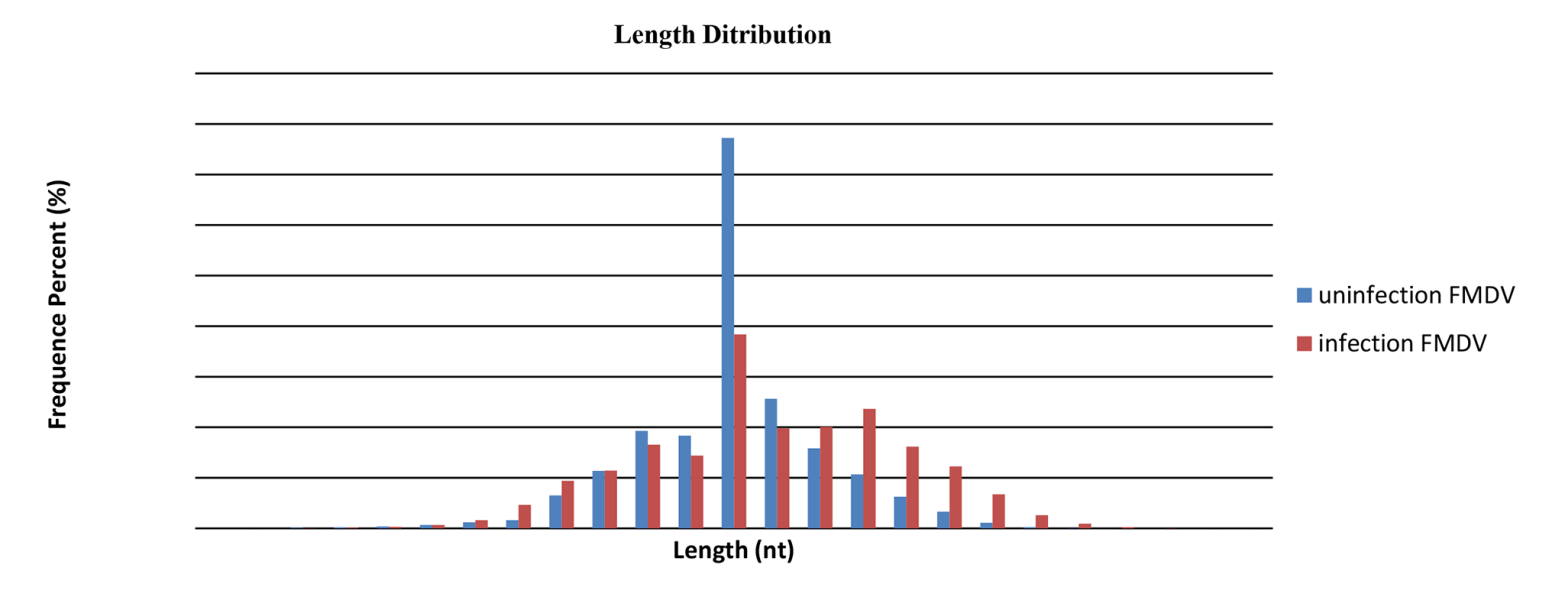
Length distribution and abundance of sequences in peak and late lactation. Sequence length distribution of clean reads based on the abundance and distinct sequences.

**Figure 2:**
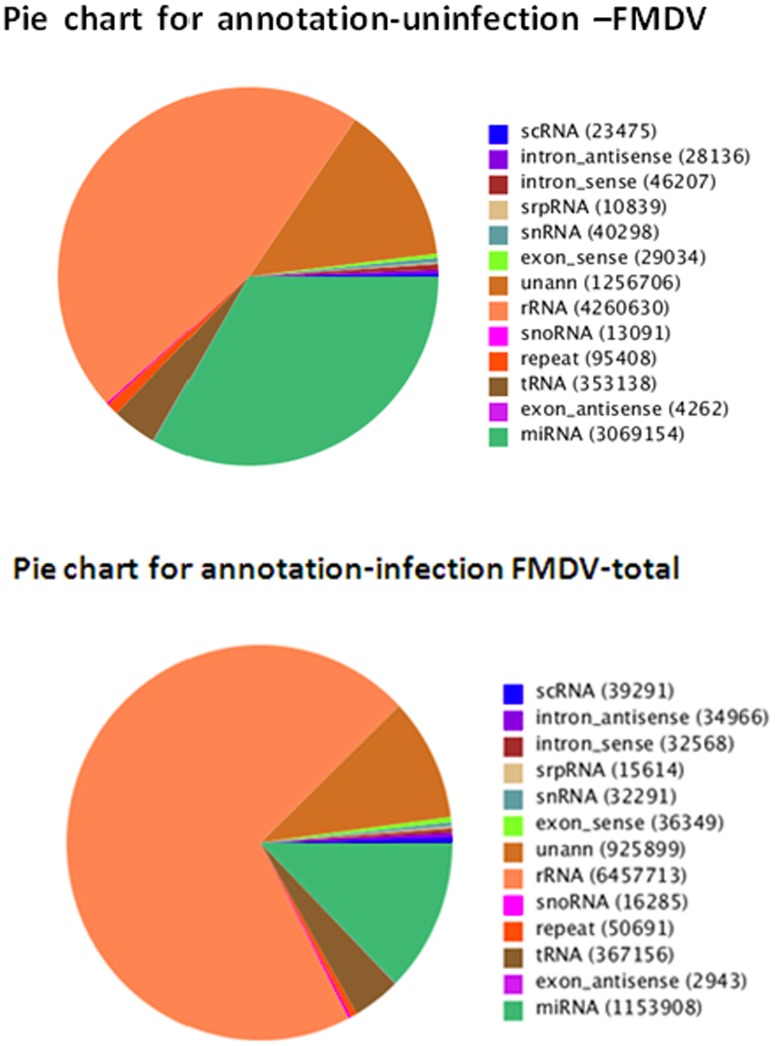
Distribution of small RNAs among different categories: infected and uninfected FMDV groups. The clean reads were annotated and classified as miRNA, rRNA, tRNA, snRNA and snoRNA in GenBank and Rfam databases, and partial reads were not annotated.

To identify miRNAs in FMDV infection in PK-15 cells, two small RNA libraries pooled from infected and uninfected groups were constructed respectively, and sequenced using an Illumina/Solexa 1G high-throughput sequencer. As a result, a total of 18,839,052 and 18,164,583 raw reads were identified in infected and uninfected groups. After removing the low quality reads, adaptors, and insufficient tags, ultimately 9,165,674 and 9,230,378 clean reads of 18∼30 nt were obtained (Table 1). The lengths of the majority of clean reads were 19∼26 nt, and a large number of the distributed sequences were 22 nt, which accounted for 19.9% and 38.62% in infected and uninfected group, and are typical of the small RNA dicer-processed products and consistent with the known 18∼25 nt range for miRNAs(Figure 1). After mapping to the *Sus crofa* genome, a total of 1839602 small RNAs sequences were provided, including 438307(accounting for 2.38%) specific sequences in FMDV-infected group, 390087 ( account for 2.12%) specific sequences in FMDV-uninfected group and 17567758(95.5%) common sequences(Table 1). All identical sequence reads were grouped together to simplify the sequencing data, with a total of 770488 unique sequences identified in two groups, which contained 324849 (account for 42.16%) specific unique sequences for infected group, 339772 (account for 42.93%) specific unique sequences for uninfected group, and 114867(14.91%) for common unique sequences (Table 1). To assess the efficiency of high-throughput sequencing for small RNA detection, all sequences were annotated and classified through alignment with GenBank and Rfam databases. All of reads were annotated and classified as miRNA, rRNA, tRNA, snRNA and snoRNA. The size distribution of the miRNAs in the infected group was less than the uninfected group was found (Figure 2). The unannotated reads were further analyzed for novel miRNA candidates.

**Table 1:** Summary of Illumina Hiseq sequencing data for small RNAs in PK-15 infected FMDV and negative groups.

Categories	Infection FMDV		NC	
	Unique sRNA	Total sRNAs	Unique sRNAs	Total sRNAs
Raw reads number		18,839,052		18,164,583
Clean reads		9,165,674		9,230,378
Perfect match to genome Percent (%)	439716 43.79%	8018737 87.49%	196095 44.0%	7815321 84.67%
Specific sequences Percent (%)	324849 42.16%	4382707 2.38%	330772 42.93%	390087 2.21%
Common Percent(%)	114867 14.91%	17567758 95.50%		
Total	324849	438207	330772	390087

### Conserved miRNAs and Differential Expression

**Figure 3:**
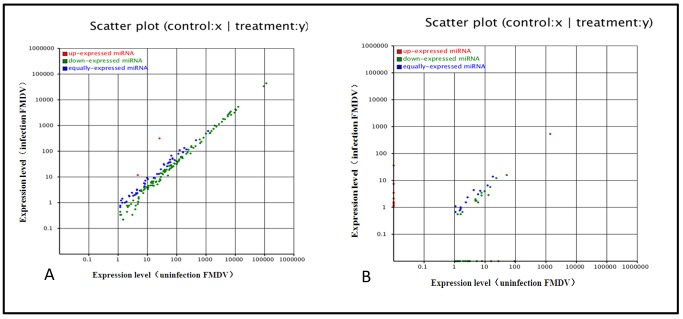
Comparison of expression levels of known miRNAs (A) and novel miRNAs (B) in infected and uninfected groups. The X- and Y-axes show the expression levels of miRNAs in the two samples. The red points represent miRNAs with ratios above 2; the blue points represent miRNAs with ratios between 1/2 and 2; the green points represent miRNAs with ratios under 1/2. Ratios = miRNA expression levels in infection/miRNA expression levels in uninfected group.

To identify the known miRNAs in our sequenced set of small RNAs, the sequences from our libraries with the repository of mature animal miRNAs in miRBase 18.0 were compared using MIREAPv0.2 software. After BLASTN searches (number mismatch ≦3) and further sequence analysis, 192556 and 196095 unique small RNAs were indicated perfect matches to known animal miRNAs deposited in miRBase 18.0, in infected and uninfected FMDV groups. According to the changes in relative miRNA abundance between the two libraries, a total of 172 differently known miRNAs were found (File S1, S2). All the different miRNAs were predominantly expressed at almost up to 1000,000 reads, suggesting abundant expression during infection. Compared with miRNA expression in uninfected group, almost all of miRNAs in infected FMDV group were significantly downregulated (P<0.01), and differentially expressed miRNAs in fold-change ranged from 0.1 to 3.0 between the two libraries (Fig. 3A, file S1). Single nucleotide polymorphism (SNP) analysis demonstrated that differences in miRNA edit positions and rates between the two libraries were identified (file S3, file S4), which may be related to FMDV infection.

### Novel miRNAs Candidates and Differential Expression

**Figure 4:**
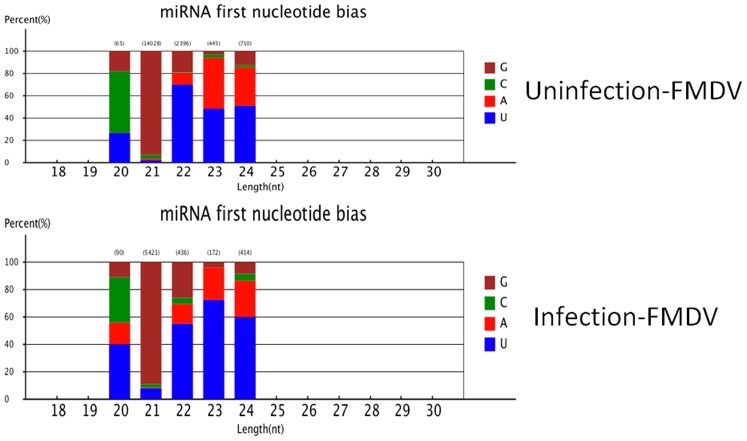
The endogenous novel miRNA exhibited significant base bias especially in 20, 22 and 24

In this study, 242885 and 268097 unannotated small RNAs were present in infected and uninfected groups. They were used to predict potentially novel miRNA candidates. Further, to determine the relationship of these small RNAs with genuine S*us scrofa* miRNA, typical secondary structures of the miRNA precursors were used to remove pseudo-miRNAs. It was predicted that 181 novel miRNAs possessed a typical stem-loop structure and free energy ranging from 25 Kcal/mol to 70 Kcal/mol (File S5, S6). According to differential analysis as discussed previously, 72 differentially expressed novel miRNA candidates were found (File S7, S8). Additionally, differential analysis showed almost all of miRNAs in the infected group were significantly downregulated (P<0.01) and differentially expressed miRNAs in fold-change ranged from 0.1 to 14.0-fold between the two libraries (Figure 3B, file S7, file S8). Additionally, novel miRNA nucleotides were detected initially at each location. Results revealed that the endogenous miRNA exhibited significant base bias, especially in 20, 23 and 24 nt miRNAs between the two groups (Fig. 4).

### Validation of Differential miRNA Expression with Quantitative RT-PCR

**Figure 5:**
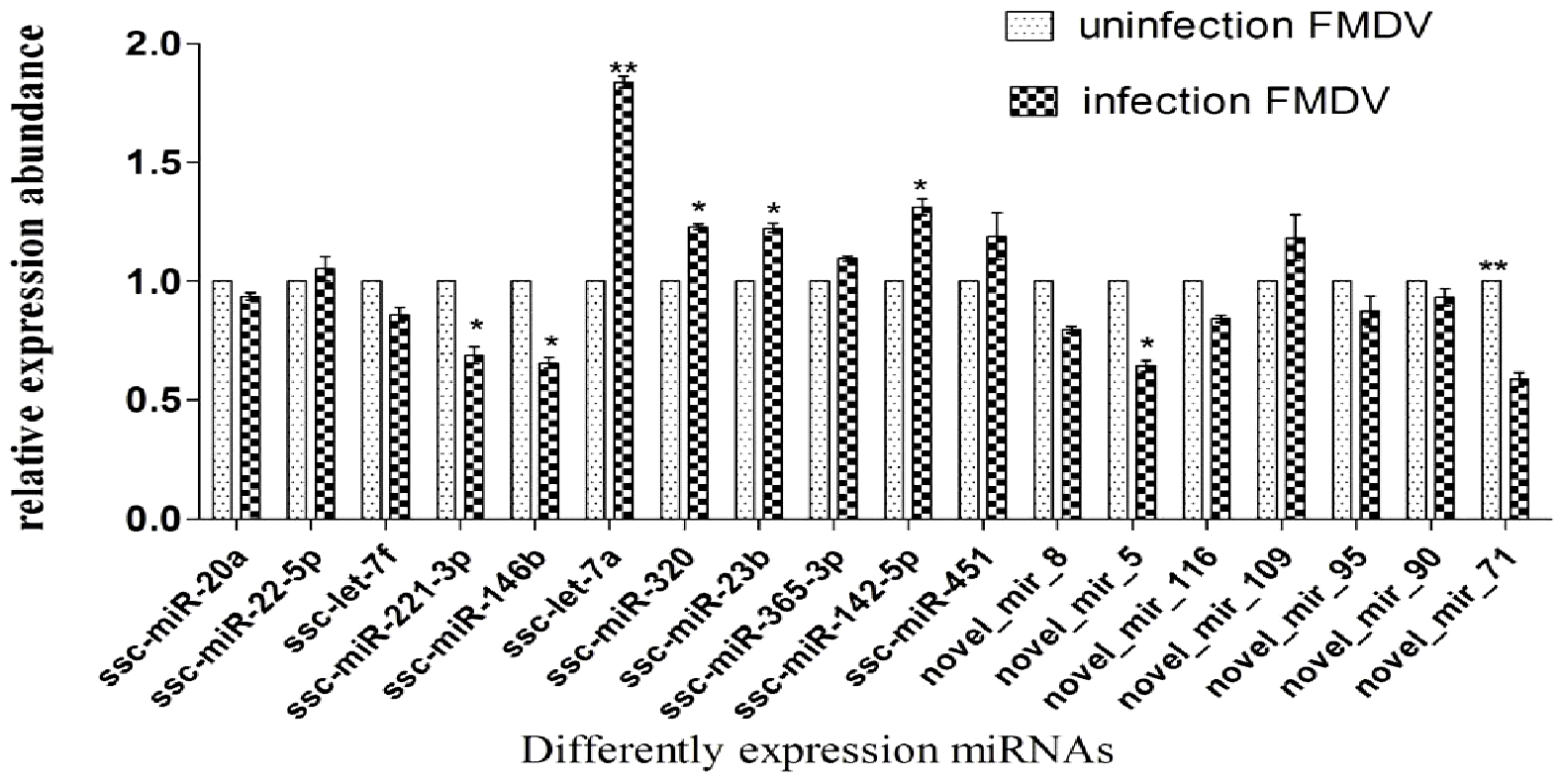
qRT-PCR validation of the identified miRNAs using Solexa sequencing technology. Real-time RT-PCR results for 11 known miRNAs in peak and late lactation. The relative quantification of expression was calculated using the 2-ΔΔ CT method after the threshold cycle (Ct) and was normalized with the Ct of U6. The relative expression levels were presented as the 2-ΔΔ CT means 6 S+SE. * represents p<0.05, ** represents p<0.01.

To confirm the reliability of the data, stem-loop qRT-PCR was used to compare differently expressed miRNAs and newly identified miRNAs. The three individuals in each group were subjected, to qRT-PCR. The 20 miRNAs differently expressed (11 known miRNAs and 9 novel miRNAs) were selected randomly and validated in infected and uninfected groups (file S1). The profile of 8 known miRNAs and 7 novel miRNAs was consistent with Solexa sequencing results, and 2 novel miRNAs were not detected (Figure 5), perhaps due to the deviation of qRT-PCR. Results indicated that ssc-miR-20a, ssc-miR-22-5p, ssc-let-7f, ssc-miR-221-3p, ssc-miR-146b, ssc-let-7a, ssc-miR-320 and ssc-miR-451 were differently expressed in two groups, and the expression levels of ssc-miR-146b, ssc-let-7a, ssc-miR-320 in uninfected group were higher than infected group.

### Target Prediction, GO and KEGG Analysis

**Figure 6:**
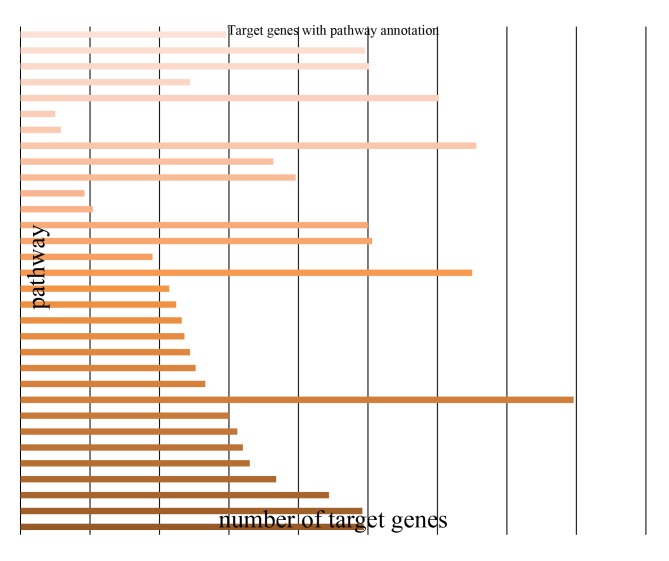
Pathway analysis of targets for known miRNAs according to KEGG function annotations.

**Figure 7:**
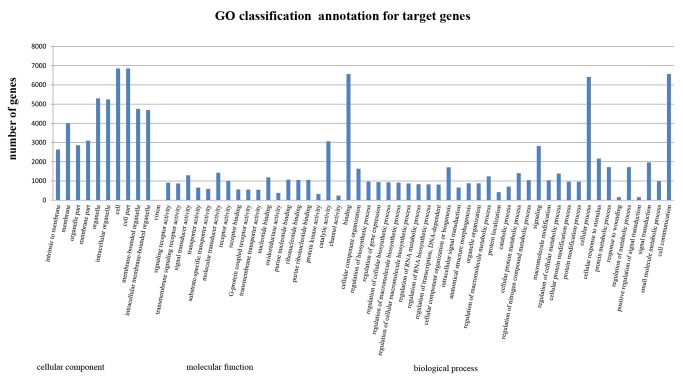
Part gene ontology classification annotated by BGI WEGO for predicted target genes. The figure shows partial GO enrichment for the predicted target genes in molecular function, cellular component and biological processes.

To understand the molecular function and biological processes of miRNAs during FMDV infection, target gene prediction of different miRNAs was performed. Determination of miRNA/mRNA interactions provided molecular insight into FMDV infection mechanisms using MIREAPv0.2 software (MATERIALS & METHODS). About 414, 359 target genes (file S9, S10) for 244 differently expressed miRNAs (known miRNA and novel miRNA) were successfully detected. The predicted targets for these miRNAs were classified according to KEGG function annotations, and identified pathways that were actively regulated by miRNAs in FMDV infection (File S11,file S12). Results showed that about 1500 predicted genes were annotated, with the genes primarily active in endocytosis, viral myocarditis, phagosome, calcium signaling, disease and signal transduction pathways associated with cellular metabolism, innate immunity and virus infection (Figure 6). Furthermore, all targets regulated by the different miRNAs were successfully classified into three modules through GO analysis(File S13, file S14).The results showed that about 8480 genes were annotated successfully, and the majority of the genes were clustered into intracellular and membrane regions (Figure 7). Molecular functional analysis showed that the functions were related to binding activity, with most of the genes involved in cellular or signaling processes.

## Discussion

In recent years, highthroughput sequencing has become a powerful strategy for identifying novel miRNAs and studying the expression profiles of miRNA in different samples. Unlike microarray technology, Solexa highthroughput sequencing sheds light on functionally novel miRNAs [29], [30]. Essentially, miRNA represents a vital component of the innate antiviral response in virus infection [31].It serves as a host gene-regulation mechanism triggered by the expression of structured miRNA molecules [11]. However, the role of host cellular miRNAs defensing against FMDV infection is still obscure. Our previous studies revealed that FMDV proliferation can reach to the maximum at the sixth hour after infection in PK-15 cells. In this study, using Solexa sequencing technology, miRNAs differentially expressed in FMDV infected and uninfected groups of PK-15 cells were identified at 6 hours point. The length distribution showed that most clean reads were primarily distributed in 19∼24 nt (Fig. 1), which is consistent with the typical size of mature mammalian miRNA from dicer-processed products. The results were consistent with previous studies [32].

However, differences in distribution existed between the two groups (Fig. 2) showed that different levels of miRNAs perform different functions during FMDV infection. The reads were aligned against the S*us scrofa* genome and annotated in GenBank and Rfam databases. It was indicated that the sequencing data in this study were highly enriched for small RNA sequences (Fig. 2). In addition to known miRNAs, functionally important novel miRNAs were initially predicted (File S5, S6), which might be undetectable using traditional methods.

The host antiviral immune response requires precise regulation [33], [34], and any aberrations are associated with potentially devastating consequences. The precise activation of antiviral immunity requires exquisite regulation of host cellular miRNAs expression [35]. Changes in host cellular miRNAs expression during FMDV infection essentially disrupts the fine mechanism controlling host antiviral immunity. Differential expression analysis showed 172 differential known miRNAs and 72 novel potential miRNAs. Compared with uninfected group, the downregulated miRNAs occupied most of the differential expressed miRNAs in infected group (Fig. 3). The mechanism of down-regulation is still unclear, although researchers revealed that downregulation of cellular miRNAs during vaccine virus infection was caused by degradation of dicer enzyme [36]. Influenza virus may also downregulate cellular miRNA expression by repressing the expression of dicer [37]. Therefore it is essential to investigate the mechanism of cellular miRNAs downregulation caused by FMDV. SNP alteration analysis of edit position and rate between the two libraries (File S3, file S4) suggested that changes in SNP may be related to FMDV infection. In addition, we found that the endogenous novel miRNA exhibited significant base bias especially in 20, 23, and 24 nt miRNAs (Fig. 4). It warrants additional research into miRNA editing mechanisms associated with FMDV infection, along with quantitative studies involvingt differential miRNA expression. The 20 differentially expressed miRNAs including 11 known miRNAs and 9 novel miRNAs, were selected randomly for validation with stem-loop qRT-PCR method. Results indicated that 18 miRNAs were validated and 11 miRNAs were consistent with our sequencing results, while 2 novel miRNAs were not obtained (Fig. 5), possibe reason is the decreased expression of the two miRNAs.

To further insights into the physiological function of miRNAs, target genes for differentially expressed miRNAs were predicted by aligning miRNA sequences to pig ESTs, following the rules of target prediction published by *Allen* [38] and *Schwab* [39] using MIREAP and targetScan 6.0 software. KEGG pathway analyses and GO annotation for target genes also offer a better understanding of the target genes at molecular, cellular and biological levels. Pathway analysis suggested that these targeted genes were involved in multiple signaling pathways, such as the cytokine receptor signaling, NOD-like receptor signaling (NLRs), and Toll-like receptor (TLRs) rand pathways (Fig. 6, file S11, S12). The activation of TLRs and NLRs triggers intracellular signaling pathways that lead to effective mechanisms in innate immunity and inflammation. Researchers found the negative regulation of this signaling during posttranscriptional regulation by miRNA-155, miRNA-146, miRNA-21, miRNA-132 [40]. As the targets of Let-7f are lymphocyte-mediated, miR-142 regulates immune response via inhibition cAMP production in CD4+, CD25+ T cells and CD4+,CD25+ TREG cells by targeting AC9 mRNA [41]. In this study, ssc-miRNA-155, ssc-miRNA-146, ssc-miR-142 and miRNA-21 (file S1) were identified. It was indicated that these miRNAs primarily related to cellular immune response to FMDV infection and FMDV replication may be indirectly regulated by these miRNAs. We also found that miRNAs are associated with cell cycle, including ssc-miR-27a, and ssc-miR-27b, which are involved in apoptosis and cell death, correlating with targets of miR-29a are miR-29b related to cell division and the cell cycle [42], [43]. GO analysis found that these genes are mainly involved in cell binding, transcriptional regulation, apoptosis regulation, and the response to stress (Fig. 7, file S13, S14).

Our future studies will aim to investigate the function of candidate miRNAs in PK-15 cells by altering their expression and investigating the effect on the immune response to FMDV infection by measuring levels of cytokine secretion and cell surface antigen expression.

## Materials and Methods

### Materials

PK-15 cells were purchased from Center for Type Culture Collection(CTCC, Wuhan, China) and cultured in modified Eagle medium (MEM, HyClone) in T-flasks (25 cm^2^) with 10% fetal bovine serum (FBS, HyClone) within 5% CO_2_ incubator. FMDV serotype Asia1 Jiangsu strain was stored at the national foot-and-mouth disease reference laboratory in China. Monolayer cells were washed three times with MEM. We added 5 mL serum-free medium containing 500 µL of 10^4^ TCID_50_/mL of Asia1 type FMDV to infetecd group of cells in T-flasks. T-flasks containing the 5 mL serum-free medium were used as control. The infected cells were harvested at 6 hours post-infection (hpi) and uninfected cells were harvested at the same time. With each group in triplicates. Samples harvested from the three groups, respectively, were mixed together and used for RNA extraction.

### Construction and Sequencing of small RNA libraries

Total RNA from each group of cells was extracted using the RNeasy Mini Kit (QIAGEN) according to the manufacturer's instructions. After purification, quality control testing for RNA was conducted using an Agilent 2100 BioAnalyzer (Agilent Technologies, Palo Alto, CA) and concentration analyzed using a Nanodrop ND-1000 (Nanodrop Technologies, Wilmington, DE). The small RNAs were size-fractionated from the RNA pool of two samples, purified by polyacrylamide gel electrophoresis to enrich for molecules in the range of 18–30 nt, and ligated to 5′- and 3′- end RNA oligonucleotide adaptors. cDNA constructs were created by RT-PCR based on the small RNAs ligated with 3′ and 5′ adaptors. The PCR products (90 bp, small RNA+adaptors) were purified with 4% agarose gels and used for sequencing with Solexa sequencing technology (Illumina HiSeq 2000, San Diego, CA, USA). All sequencing was carried out at the Beijing Genomics Institute (BGI), Shenzhen.

### Analysis of Sequence Data

Low quality reads were filtered according to the base quality value. The clean reads were mapped to the *sus scrofa* genome to analyze their expression and distribution using SOAPv1.11 software [44]. All validated sequences were obtained for further analysis. The sequences were aligned against the known miRNAs precursors and mature miRNAs deposited in the miRBase 18.0 to identify conserved miRNAs. The unannotated sequences were used to predict potentially novel miRNA candidates. Mfold3.2 software was used to analyze the RNA secondary structures [45], and MIREAPv0.2 software (https://sourceforge.net/projects/mireap/) [46] was used to predict the potential miRNA candidates according to established rules [47], [48].

### Differentially Expressed miRNAs in infected and uninfected PK-15 cells

The random variance model corrected t-test (RVM-T test) was used to calculate the significance level (P-value) of miRNA expression and the false positive rate (FDR), and miRNAs differentially expressed in infected and uninfected PK-15 cells were identified. The UCSC genome database was used to identify pri-miRNA sequences and all differentially expressed miRNAs were compared with the Sanger database to determine the specific location of pri-miRNAs in porcine genome. The presence of transcription factor binding sequences in pri-miRNA regulatory regions was used to indicate transcription factors that may regulate their transcription. This result was used to construct a regulatory network of transcription factors and miRNA molecules.

### Prediction of Differential Expression of miRNA Target Genes

The target genes for differentially expressed miRNAs (known as miRNAs, novel miRNAs) were predicted by aligning the Sus scrofa EST database in NCBI, according to previously established rules [38], [39].

### GO Annotation and KEGG Pathway Analysis of Target Genes

According to established algorithm [48], InterProScan [49] and BGI WEGO (http://wego.genomics.org.cn/cgi-bin/wego/index.pl) [50] were used to perform GO annotation and enrichment analysis for three ontologies, molecular function, cellular component and biological process. The genes with FDR ≦0.5 were considered as significantly enriched in target gene candidates. Target genes involved in significant metabolic or signal transduction pathways were analyzed to decipher the KEGG (Kyoto Encyclopedia of Genes and Genomes, http://www.genome.jp/kegg/) [51].

### Quantitation of Target miRNA in Infected and Uninfected PK-15 cells

Differentially expressed miRNAs were validated using relative real-time quantitative RT-PCR according to the manufacturer's protocol. The stem-loop quantitative real-time PCR (qRT-PCR) was performed using ABI RPISM 7500 sequence detection system and SYBR Green qPCR SuperMix (Invitrogen)..RNA was treated with DNaseI (Promega) and cDNAs were synthesized using superscript III transcriptase (Invitrogen) with miRNA-specific forward primer and universal reverse primer. The miRNA-specific stem-loop RT primers were designed (Table 2) and synthesized with the software primer 5.0. Housekeeping gene U6 was used as the internal reference. Briefly, 20 ul PCR reaction contained about 5 ng miRNA first-strand cDNA synthesis, 10 ul 2× miRcute miRNA premix, and 200 nM of each primer. The reactions were mixed gently and incubated at 94°C for 2 min, followed by 40 cycles of 95°C 15 s, 60°C 32 s and subsequent elongation at 72°C for 5 min. All samples were performed in triplicates. PCR efficiency calculations were based on the slopes of the standard curves. The absolute amount of each miRNA was calculated using the 2^−ΔΔCT^ method [52] according to the standard curve. Each miRNA level was expressed as 2^−ΔΔCT^ mean+SE. One-way ANOVA was used to examine the significance of differential expression level in each known/novel miRNA between infected and uninfected groups, and the difference was considered as significant when P<0.05.

**Table 2:** Summary of miRNA primers used in real-time qRT-PCR.

miRNAs name	Target Sequence	Primer(5′-3′)
ssc-miR-20a	TAAAGTGCTTATAGTGCAGGTA	ACACTCCAGCTGGGTAAAGTGCTTAGTGC
ssc-miR-22-5p	AGTTCTTCAGTGGCAAGCTTTA	ACACTCCAGCTGGGATCACATTGCCAGGGAT
ssc-let-7f	TGAGGTAGTAGATTGTATAGTT	ACACTCCAGCTGGGTGAGGTAGTAGGTTGTA
ssc-miR-221-3p	AGCTACATTGTCTGCTGGGTTT	ACACTCCAGCTGGGAGCTACATTGTCTGCTG
ssc-miR-146b	TGAGAACTGAATTCCATAGGC	ACACTCCAGCTGGGTGAGAACTGAATTCCAT
sc-let-7a	TGAGGTAGTAGGTTGTATAGTT	ACACTCCAGCTGGGGTGAGGTAGTAGGTTGTA
ssc-miR-320	AAAAGCTGGGTTGAGAGGGCGAA	ACACTCCAGACTGGGAAAAGCTGGGTTGAGAG
ssc-miR-23b	ATCACATTGCCAGGGATTACCA	ACACTCCAGCTGGGATCACATTGCCAGGGAT
ssc-miR-365-3p	TAATGCCCCTAAAAATCCTTAT	ACACTCCAGCTGGGTAATGCCCCTAAAAATC
sc-miR-142-5p	CATAAAGTAGAAAGCACTACT	ACACTCCAGCTGGGCATAAAGTAGAAAGCACT
ssc-miR-451	AAACCGTTACCATTACTGAGTT	ACACTCCAGCTGGGAAACCGTTACCATTACTGA
Novel 0008-3p	AGAGGAGAGGCTACCACCACCA	ACACTCCAGCTGGGAAACCGTTACCATTACGGT
Novel 0011-3p	AGAGGAGAGGCTACCACCACCA	ACACTCCAGCTGGGAGAGGAGAGGCTACCACC
Novel 0016-5p	GAGAGATCAGAGGCGCAGAGT	ACACTCCAGCTGGGGAGAGATCAGAGTCTCA
Novel 0025-5p	GAGGTGCTGCAGGAGGTGGGCTCT	ACACTCCAGCTGGGGAGGTGCTGACCGAGA
Novel 0063-5p	GGGGAACTCCCAGACCAGCTTC	ACACTCCAGCTGGGGGGGAACTCCCATTC
Novel 0093-3p	TGGATTGTTCTCCAACCTGGCTCT	ACACTCCAGCTGGGTGGATTGTTCTCCAAGAGA
Novel 0094-3p	TGGATTGTTCTCCAACCTGGCTCT	ACACTCCAGCTGGGTGGATTGTTCTCCAAACGA
U6-F		ATCGGTTGGCAAACGTTTC
U6-R		TGCGCAGTGGTTTTTGA

## Supporting Information

File S1 **Differentially expressed known miRNAs.**
(XLSX)

File S2 **Sequences of different known miRNAs.**
(XLSX)

File S3 **Characteristics of miRNAs edit in uninfected FDMV group.**
(XLSX)

File S4 **Characteristics of miRNAs edit in infected FDMV group.**
(XLSX)

File S5 **Typical stem-loop structure and free energy range of novel miRNAs candidates in uninfected FMDV group.**
(XLSX)

File S6 **Typical stem-loop structure and free energy ranging of novel miRNAs candidates in infected FMDV group.**
(XLSX)

File S7 **Differentially expressed novel miRNA candidates.**
(XLSX)

File S8 **Sequences of different novel miRNA candidates.**
(XLSX)

File S9 **Target genes for different known miRNAs.**
(XLS)

File S10 **Target genes for novel miRNA candidates.**
(XLS)

File S11 **KEGG Pathway annotations for the predicted target genes for different known miRNAs.**
(XLSX)

File S12 **KEGG Pathway annotations for the predicted target genes for different novel miRNA candidates.**
(XLSX)

File S13 **GO annotations for the predicted target genes for different known miRNAs.**
(XLSX)

File S14 **GO annotations for the predicted target genes for different novel miRNA candidates.**
(XLSX)
