# Correction: Identification and Analysis of Differential miRNAs in PK-15 Cells after Foot-and-Mouth Disease Virus Infection

**DOI:** 10.1371/journal.pone.0101635

**Published:** 2014-06-24

**Authors:** The PLOS ONE Staff

There is an error in the first name of the co-author, Dr. Xian-Tao Liu. The correct name for this co-author is: Dr. Xiang-Tao Liu.
